# Early identification of infectious complications in pediatric burn patients: a prospective cohort study

**DOI:** 10.1186/s12887-026-06867-7

**Published:** 2026-04-20

**Authors:** Bohumil Bakalář, Robert Zajíček, David Frejlach, Václava Adámková, Terezie Poláčková, Petra Bořilová Linhartová, Marta Fridrichová, Helena Lahoda Brodská

**Affiliations:** 1 Prague Burn Centre, The Third Medical Faculty, Charles University and The Kralovske Vinohrady University Hospital, Prague, Czech Republic; 2 Department of Anesthesia and Intensive Care, The Third Medical Faculty, Charles University and The Kralovske Vinohrady University Hospital, Prague, Czech Republic; 3 Institute of Medical Biochemistry and Laboratory Diagnostics, The First Faculty of Medicine of Charles University and The General University Hospital, Prague, Czech Republic; 4 RECETOX, Faculty of Science, Masaryk University, Kotlarska 2, Brno, Czech Republic; 5 Department of Microbiology, Central Laboratories, The Third Medical Faculty, Charles University and The Kralovske Vinohrady University Hospital, Prague, Czech Republic

**Keywords:** Pediatric burns, Infection, Biomarkers, Intensive care infection score, Antimicrobial stewardship, Pediatric intensive care

## Abstract

**Objective:**

To assess early identification of infectious complications in pediatric burn patients and to evaluate the performance of the Intensive Care Infection Score (ICIS) in comparison with conventional inflammatory biomarkers.

**Design:**

Prospective observational cohort study.

**Setting:**

Tertiary pediatric burn intensive care unit.

**Patients:**

Children admitted within 24 h of acute burn injury and requiring intensive care for ≥ 3 days.

**Interventions:**

None.

**Measurements and main results:**

Sixty-nine children were enrolled; 18 (26%) experienced at least one infectious episode. Infection was defined using a composite reference standard that included clinical diagnosis, microbiological findings, and initiation of targeted antibiotic therapy. ICIS was calculated from extended complete blood count parameters obtained during routine clinical care. Receiver operating characteristic analysis demonstrated higher discriminative performance of ICIS compared with C-reactive protein and procalcitonin in this cohort. The area under the curve for ICIS was 0.93, compared with 0.71 and 0.72 for C-reactive protein and procalcitonin, respectively. At a cutoff value of ≥4, ICIS demonstrated very high sensitivity and negative predictive value in this cohort, with no false-negative observations, and a specificity of 0.70.

**Conclusions:**

In pediatric burn patients, ICIS demonstrated high diagnostic accuracy in this cohort and improved discrimination compared with C-reactive protein and procalcitonin, particularly as a rule-out test. These findings provide novel evidence on the application of ICIS in a pediatric burn population. As ICIS is derived from routine blood counts without additional sampling or direct laboratory cost, it may represent a practical tool to support early clinical decision-making and antimicrobial stewardship in pediatric burn care.

**Supplementary Information:**

The online version contains supplementary material available at 10.1186/s12887-026-06867-7.

## Information box: What is known

Early differentiation between infection and sterile inflammation in pediatric burn patients is challenging because burn injury itself triggers a systemic inflammatory response. Conventional biomarkers such as C-reactive protein and procalcitonin are widely used but have limited specificity and variable diagnostic performance in children. The Intensive Care Infection Score (ICIS), derived from extended complete blood count parameters, has shown good diagnostic accuracy for infection in adult intensive care populations, but evidence in pediatric patients, particularly those with burn injuries, has been lacking.

## Information box: What is new

This prospective cohort study suggests that ICIS may have high diagnostic accuracy for early infection detection in pediatric burn patients and showed higher discriminative performance than commonly used biomarkers in this cohort. At a cutoff value of ≥ 4, ICIS demonstrated very high sensitivity and negative predictive value in this cohort, with no false-negative observations. Because ICIS is calculated automatically from routine blood counts without additional sampling or cost, it represents a practical decision-support tool that may help safely rule out infection and support antimicrobial stewardship in pediatric intensive care units.

## Introduction

Burn injuries in children pose significant clinical challenges due to their systemic effects and high risk of infection. Even minor burns can trigger systemic inflammatory response syndrome (SIRS) [1], complicating the distinction between sterile inflammation and sepsis in the pediatric intensive care unit (PICU). Accurate differentiation is essential to avoid unnecessary antibiotic use and improve clinical outcomes. Early infection detection supports antimicrobial stewardship and reduces unnecessary antimicrobial exposure.

Common biomarkers such as C-reactive protein (CRP), procalcitonin (PCT), and white blood cell count (WBC) assist clinicians; however, their interpretation in children is complicated by age-related variability [2, 3]. Consequently, no universally accepted standard exists for early infection detection in pediatric burn patients.

The Intensive Care Infection Score (ICIS), a composite index derived from routine complete blood count (CBC) parameters, has demonstrated utility in adult critical care for distinguishing infectious from noninfectious conditions [4–6]. The ICIS is calculated using five parameters: the number of neutrophil segments, the number of immature granulocytes, the mean fluorescence intensity of neutrophil segments, the difference in hemoglobin concentration of mature and young cells, and the number of antibody-secreting lymphocytes. These parameters reflect early innate immune activation. The maximum ICIS value is 20.

Evidence in pediatric populations, particularly those with burn injuries, remains scarce.

This study aimed to assess early identification of infectious complications in pediatric burn patients and to evaluate the performance of ICIS in comparison with conventional inflammatory biomarkers, including CRP, PCT, WBC, calprotectin (CAL), presepsin (PRE), and lipopolysaccharide-binding protein (LBP). To date, studies evaluating ICIS have been limited to adult populations and selected clinical settings. Data on the performance of ICIS in pediatric patients, and particularly in pediatric burn populations, are lacking.

## Materials and methods

A single-center, prospective observational study was conducted at a national pediatric burn center in the Czech Republic.

### Participants

Children with thermal injuries admitted to the PICU within 24 h of injury between March 17, 2021, and May 9, 2022, were eligible. Inclusion criteria included an ICU stay of ≥ 3 days. Exclusion criteria included: use of immunosuppressive or chemotherapy agents; abnormal hematologic indices on admission (anemia: hemoglobin ≤ 95 g/L for children < 6 years of age or ≤ 100 g/L for older children; leukopenia: WBC ≤ 3.9 × 10⁹/L; thrombocytopenia: platelet count ≤ 149 × 10⁹/L); or refusal of informed consent.

### Procedures

Blood samples were collected at ICU admission and every three days thereafter, with additional samples if infection was suspected. Infection surveillance was performed daily. Assessed biomarkers included ICIS, WBC, CRP, PCT, PRE, CAL, and LBP.

ICIS is derived from five extended hematologic parameters reflecting innate immune activation [7]. ICIS calculation requires a compatible hematology analyzer equipped with manufacturer-specific flow cytometry–based technology and a dedicated research-use-only (RUO) software module. At present, ICIS is available only on selected analyzers and is not universally implemented in routine clinical practice. In this study, ICIS values were generated retrospectively and were not used to guide clinical decision-making.

CRP and PCT were measured using automated immunoassays; PRE and CAL by chemiluminescent methods; and LBP using immunoassay (see Supplementary Methods in the Supplementary Appendix). Reference ranges followed manufacturer specifications.

Classification of patients into an infection and no-infection group was based on clinical diagnosis, initiation of targeted antibiotic therapy, and microbiological or imaging evidence, as assessed by two senior intensivists blinded to ICIS results. In cases of disagreement, classification was resolved by consensus discussion.

Comprehensive microbiological testing was performed according to clinical indications as part of standard care. Depending on the presumed or suspected source of infection, the following microbiological investigations included central and peripheral blood cultures, urine cultures, bronchoalveolar lavage, nasal lavage, pleural effusion, and wound exudate. Additionally, wound swabs were collected for microbial examination.

### Statistical analysis

Analyses were performed at the observation level to reflect real-world clinical decision-making in burn care. Group comparisons of biomarker values between infected and non-infected observations were performed using the two-sided Wilcoxon rank-sum test (Mann–Whitney). To control the family-wise rate of false discoveries across predefined hypothesis families, the Benjamini–Hochberg procedure was applied with an FDR of 0.05; statistical significance was based on adjusted p-values (q-values). Correlations between biomarkers, as well as between biomarkers and total body surface area burned (TBSA), were estimated using Kendall’s tau-b, which is robust to outliers and ties. For diagnostic discrimination, receiver operating characteristic (ROC) analyses were performed for each biomarker. The area under the ROC curve (AUC) was reported with 95% confidence intervals obtained via cluster bootstrap (resampling at the patient level) to account for repeated measures. Optimal thresholds were determined using the Youden index (maximizing sensitivity + specificity − 1). At the optimal threshold, sensitivity, specificity, positive predictive value (PPV), and negative predictive value (NPV) were reported with 95% confidence intervals calculated using Wilson’s method, together with confusion-matrix counts (TP, FP, TN, FN).

## Results

### Study population

**Fig. 1:**
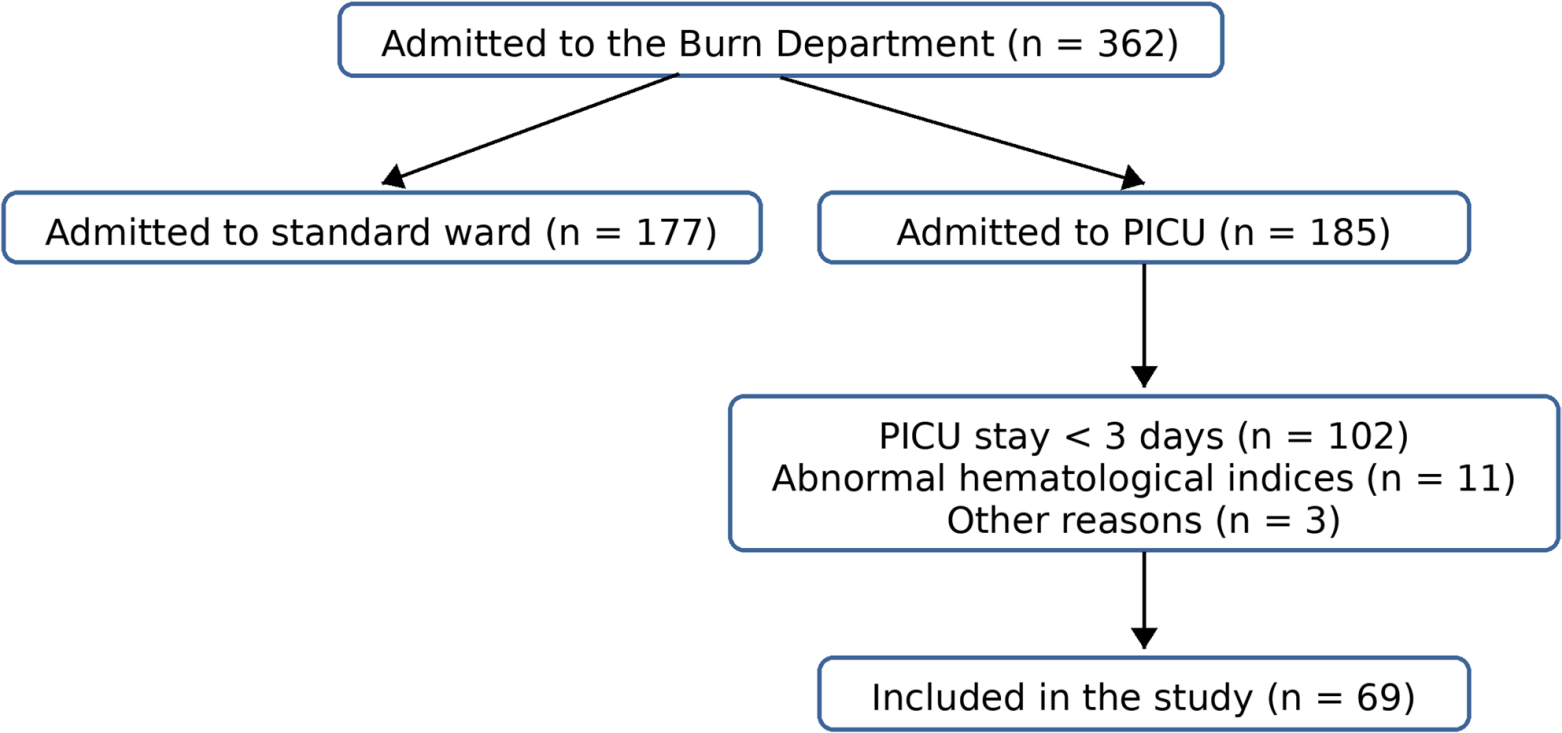
Patient selection flowchart

During the study period, 69 children admitted to the PICU within 24 h of acute burn injury were enrolled (Fig. 1) and retrospectively classified into infection or no-infection groups. The median age was 18 months (IQR, 15–27). Eighteen patients (26.1%) experienced at least one episode meeting infection criteria, whereas 51 (73.9%) remained in the non-infected category during the observation period. A total of 145 observations (samples) were analyzed across 69 patients.

Baseline characteristics were broadly similar between groups (Table 1). There were no significant differences in age, burn size (total body surface area [TBSA]), length of stay (LOS), or sex distribution between infected and non-infected patients.

**Table 1:** Baseline characteristics

Parameter	Infected patients (*n* = 18)	Non-infected patients (*n* = 51)	*p*-value
Age, months	20.5 (15.3–24.8)	18.0 (14.5–27.0)	0.56
TBSA, %	13.5 (12.0–21.5)	12.0 (8.0–15.5)	0.87
Sex, male/female	10 (55.6%) / 8 (44.4%)	32 (60.9%) / 19 (39.1%)	0.86
Length of stay, days	11.5 (10.0–20.8)	10.0 (7.0–16.8)	0.93

Data are presented as median (interquartile range) or number (percentage). TBSA, total body surface area

Most participants were between 1 and 3 years of age (*n* = 51), with 15 infections recorded in this age group. Infants (< 1 year) accounted for 6 patients (1 infection), and children older than 3 years accounted for 12 patients (2 infections) (Table 2).

**Table 2:** Age groups

Age group (years)	*n* (% of total cohort)	Infected patients, *n* (% within age group)
< 1	6 (8.7%)	1 (16.7%)
1–3	51 (73.9%)	15 (29.4%)
> 3	12 (17.4%)	2 (16.7%)

Percentages refer to the total cohort for N and to the respective age group for infected patients

Infectious complications were predominantly localized rather than systemic. Burn wound infections represented the most frequent infectious site, followed by respiratory, urinary tract, and bloodstream infections (Table 3). Several patients experienced infections at more than one anatomical site.

**Table 3:** Types of infectious complications observed during the study period

Site of infection	*n* (observations)
Burn wound infection	18
Respiratory tract infection (BAL)	9
Urinary tract infection	7
Bloodstream infection	5

Some patients experienced infections involving more than one anatomical site

### Diagnostic performance of biomarkers

**Fig. 2:**
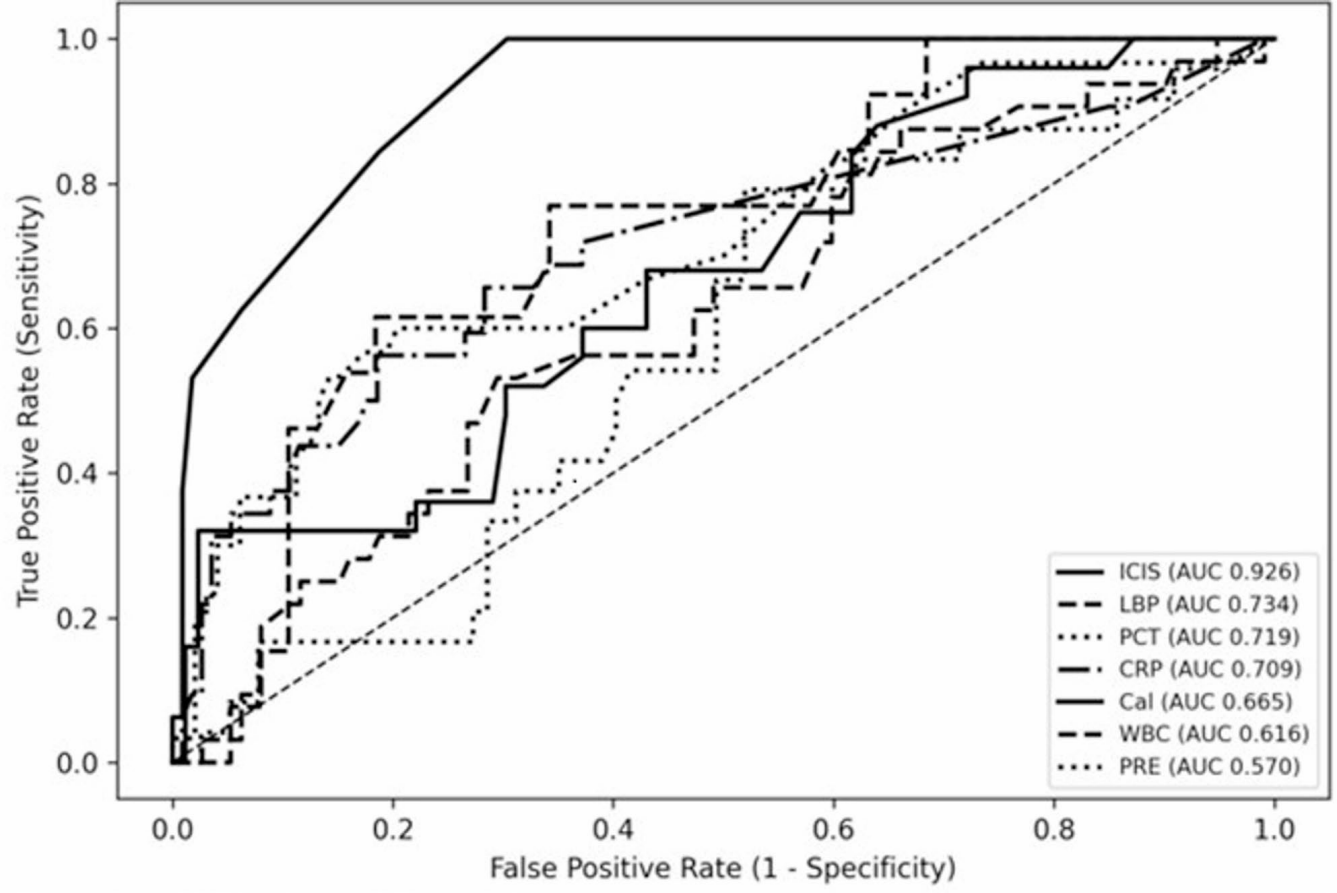
ROC curves for all study biomarkers. Lines represent individual biomarkers; AUC values are shown in the legend. ICIS, Intensive Care Infection Score; CRP, C-reactive protein; PCT, procalcitonin; WBC, white blood cell count; PRE, presepsin; CAL, calprotectin; LBP, lipopolysaccharide-binding protein

Receiver operating characteristic analyses derived from repeated observations demonstrated strong rule-out capability of ICIS in this cohort at a threshold of ≥ 4 (Fig. 2). Compared with conventional biomarkers, ICIS showed the highest overall diagnostic performance, with high sensitivity and negative predictive value, supporting its use as a rule-out test for infection. In contrast, C-reactive protein and procalcitonin demonstrated lower discriminative performance. Eighteen observations were excluded because of missing biomarker values.

Diagnostic performance of all biomarkers, including WBC, PRE, CAL, and LBP, is provided in Supplementary Table S1. Comparisons of biomarker levels between infected and non-infected observations are provided in Supplementary Table S2.

Correlations between study biomarkers and TBSA were not significant (Supplementary Figure S1 and Supplementary Table S3 and S4).

Infected observations (*n* = 32) were further analyzed according to the timing of ICIS sampling in relation to antibiotic therapy. In 6 of 32 infected observations (19%), ICIS sampling was performed before initiation of antibiotic treatment, whereas in the remaining cases ICIS was obtained during ongoing antimicrobial therapy. Importantly, ICIS values were not used to guide antibiotic initiation, which was based exclusively on clinical assessment and standard diagnostic work-up.

A detailed overview of microbiologically documented infectious episodes is provided in Supplementary Table S5.

In a sensitivity analysis restricted to the first available observation per patient, performed to address potential bias related to repeated sampling, diagnostic performance estimates remained consistent with the primary analysis (Supplementary Table S6).

## Discussion

In this prospective pediatric burn cohort, early identification of infectious complications remained challenging, and ICIS showed higher discriminative performance than CRP and PCT, particularly as a rule-out test. Using a cutoff of ≥ 4, ICIS demonstrated very high sensitivity and negative predictive value in this cohort, with no false-negative observations, albeit at the expense of lower specificity and a higher rate of false-positive results. This trade-off is clinically acceptable in pediatric critical care, where failure to detect early infection can have substantial consequences. Importantly, sensitivity analyses restricted to the first sample per patient yielded comparable results, supporting the robustness of the findings despite repeated measurements in the primary analysis. Although observation-level analysis reflects real-world clinical decision-making, it may overestimate diagnostic performance due to within-patient correlation.

To our knowledge, this study represents the first prospective evaluation of ICIS in a pediatric burn population. Although the overall incidence of infection may appear high relative to burn size, most infectious episodes represented localized burn wound infections rather than systemic sepsis or septic shock. This pattern is typical for pediatric burn patients requiring intensive care and prolonged wound management and helps explain the observed infection frequency despite relatively modest TBSA involvement.

Importantly, the optimal ICIS cutoff (≥ 4) was identical to that reported in adult ICU cohorts, supporting both biological plausibility and external validity. Our findings are consistent with reports from mixed adult ICU populations in which ICIS demonstrated high discriminative performance and higher AUCs compared with CRP and PCT, supporting its potential utility for early diagnostic triage and antimicrobial stewardship initiatives [5, 8]. At the same time, conventional biomarkers such as CRP and PCT have shown variable sensitivity and specificity in pediatric burns across the literature [9], underscoring the difficulty of distinguishing sterile inflammation from infection in this setting. Recent reviews also emphasize that no single biomarker is sufficient for the diagnosis of burn-related sepsis and that multimarker approaches may be required [10].

Current guidelines, including the *American Burn Association consensus criteria* for sepsis in burn patients [11, 12] and pediatric critical care recommendations [13], emphasize early recognition and timely intervention to prevent progression to septic shock and organ dysfunction. However, these guidelines rely heavily on clinical judgment and conventional biomarkers, which are frequently confounded by burn-related inflammation. ICIS should be viewed as a decision-support tool rather than as a standalone diagnostic test. Incorporating ICIS into this framework could strengthen diagnostic confidence and support adherence to antimicrobial stewardship principles.

### Clinical implications

ICIS is calculated automatically using flow cytometry–derived extended hematologic parameters obtained from routine complete blood counts. Although its calculation currently requires dedicated analyzer-specific technology, it does not require additional blood volume, specialized assays, or supplementary sampling beyond standard care. This feature may facilitate timely availability of results and support clinical assessment in settings where rapid access to advanced biomarkers is limited.

The strong negative predictive value observed at the selected cutoff suggests that ICIS may be particularly useful as a rule-out tool, helping clinicians identify pediatric burn patients at low risk of infection and potentially avoid unnecessary initiation or escalation of antibiotic therapy. Conversely, elevated ICIS values may prompt closer clinical evaluation, targeted microbiological sampling, and imaging in accordance with established pediatric burn care guidelines, rather than serving as a standalone trigger for antimicrobial treatment.

### Limitations

This single-center observational study has several limitations. First, the modest sample size limits statistical precision, and repeated measurements per patient may have inflated the number of observations and potentially overestimated diagnostic performance. Second, infection classification relied on a pragmatic composite of clinical and microbiological criteria; although reflective of real-world practice, this approach may introduce classification bias, particularly due to the inclusion of antibiotic initiation as a criterion, and some degree of misclassification cannot be excluded. Third, a limited number of observations were excluded because of missing biomarker values, which may have introduced selection bias. A limited proportion of infected observations were obtained prior to antibiotic initiation, which may have influenced biomarker levels and diagnostic performance estimates. In addition, exclusion of patients with marked baseline cytopenias was applied to ensure validity of ICIS calculation but may have limited inclusion of patients with severe hematologic derangements.

ICIS is currently available only on selected hematology analyzers and requires dedicated manufacturer-specific hardware and research-use-only (RUO) software, which may limit its immediate applicability across all pediatric burn centers. Furthermore, diagnostic thresholds derived in this cohort may not be directly generalizable to other pediatric burn units or laboratory platforms.

### Future directions

Multicenter validation in pediatric burn cohorts is warranted to confirm optimal thresholds and to evaluate ICIS kinetics in relation to clinical events and conventional markers. Age-stratified cutoffs, integration with antimicrobial stewardship pathways, and assessment of patient-centered outcomes (antimicrobial exposure, length of stay, and complications) should be prioritized.

## Conclusions

ICIS may provide a useful rule-out tool for infection in pediatric burn patients; however, these findings should be interpreted in the context of the modest sample size and require validation in larger, multicenter cohorts.

## Supplementary Information

Supplementary Material 1.

## Data Availability

The datasets used and/or analyzed during the current study are available from the corresponding author on reasonable request.
