# Supplementary material for: Early identification of infectious complications in pediatric burn patients: a prospective cohort study
Source: BMC Pediatr. 2026 Apr 20;26:511. doi: 10.1186/s12887-026-06867-7 (PMC13231677; doi:10.1186/s12887-026-06867-7)
Supplement: Supplementary file 1 — Supplementary Material 1. [file 12887_2026_6867_MOESM1_ESM.docx]

**Supplementary Appendix**

**Early identification of infectious complications in pediatric burn patients: a prospective cohort study**

Bakalář B., Lahoda Brodská H., Frejlach D., Adámková V., Poláčková T., Bořilová Linhartová P., Fridrichová M., Zajíček R.

This supplemental material is provided by the authors to give readers additional methodological details and extended results.

**Table of Contents**

Supplementary Methods .................................................................................................................................... 2

Supplementary Table S1. ROC analysis, Youden cut-off and diagnostic performance with 95% CI .............. 3

Supplementary Table S2. Comparison of biomarker levels between infected and non-infected observations .. 4

Supplementary Table S3. Kendall’s tau-b correlations between biomarkers with FDR correction ................... 5

Supplementary Table S4. Kendall’s tau-b correlations between biomarkers and TBSA with FDR correction .. 6

Supplementary Figure S1. Correlation among biomarkers and TBSA ................................................................ 7

Supplementary Table S5. Detailed overview of infectious episodes …………………………………….…….. 8

Supplementary Table S6. Sensitivity analysis restricted to the first sample per patient ………………………... 9

**Supplementary Methods**

*Analytical methods*. PCT was determined by chemiluminescence immunoassay using an automated analyser DXI Beckman Coulter (PCT reference values <0.05 µg/L). CRP was determined by immunoturbidimetric method using an automated analyser DXI Beckman Coulter (CRP reference values <0.5 mg/L). Presepsin was measured using the PATHFAST™ analytic system (Mitsubishi) based on chemiluminescent enzymatic immunoanalysis (PES reference values < 160 ng/L). Calprotectin was measured using the electrochemiluminescent assays QUANTA Flash® Circulating Calprotectin by Inova Diagnostics in the Cobas (Roche Diagnostics) analyser (CAL reference values < 3.5 mg/L). Lipopolysaccharide binding protein was determined by chemiluminescence immunoassay using an automated analyser Atelica Siemens (LBP reference values 2.3-8.3 µg/L).

Blood samples for WBC and ICIS assessment were collected in K3EDTA tubes and analyzed immediately using a modified fluorescence flow hematology analyzer with fully automated gating (Sysmex, Kobe, Japan). A detailed technical description of ICIS components is provided for completeness. ICIS is a composite score reflecting innate immune activation and consists of five routinely available blood cell–derived parameters: mean fluorescence intensity of mature neutrophils, hemoglobin concentration difference between reticulocytes and mature erythrocytes, absolute segmented neutrophil count, antibody-secreting lymphocyte count, and accurate immature granulocyte count. All parameters are obtained without sample preparation using routine fluorescence flow cytometry based on nucleic acid–specific reagents and cell membrane–targeting surfactant reagents, with forward and side scatter used to assess cell size and intracellular complexity. The ICIS ranges from 0 to 20, calculated as the sum of weighted values of the five components.

**Supplementary Table S1.** ROC analysis, Youden cut-off and diagnostic performance with 95% CI

| ***Marker*** | ***N*** | ***AUC*** | ***AUC***  ***(95% CI)*** | ***Cut-off*** | ***Sensitivity*** | ***Sensitivity (95% CI)*** | ***Specificity*** | ***Specificity (95% CI)*** | ***PPV*** | ***PPV***  ***(95% CI)*** | ***NPV*** | ***NPV***  ***(95% CI)*** | ***TP*** | ***FP*** | ***TN*** | ***FN*** |
| --- | --- | --- | --- | --- | --- | --- | --- | --- | --- | --- | --- | --- | --- | --- | --- | --- |
| ***ICIS*** | 144 | 0.926 | (0.885–0.962) | 4.00 | 1.00 | (0.893–1.000) | 0.70 | (0.606–0.774) | 0.48 | (0.368–0.603) | 1.00 | (0.953–1.000) | 32 | 34 | 78 | 0 |
| ***LBP*** | 51 | 0.734 | (0.541–0.870) | 19.30  mg/L | 0.62 | (0.355–0.823) | 0.82 | (0.666–0.908) | 0.53 | (0.301–0.752) | 0.86 | (0.713–0.939) | 8 | 7 | 31 | 5 |
| ***PCT*** | 128 | 0.719 | (0.592–0.824) | 0.17  µg/L | 0.60 | (0.423–0.754) | 0.80 | (0.706–0.864) | 0.47 | (0.325–0.627) | 0.87 | (0.781–0.922) | 18 | 20 | 78 | 12 |
| ***CRP*** | 145 | 0.709 | (0.601–0.821) | 18.10  mg/L | 0.56 | (0.393–0.718) | 0.81 | (0.733–0.875) | 0.46 | (0.316–0.614) | 0.87 | (0.790–0.920) | 18 | 21 | 92 | 14 |
| ***CAL*** | 111 | 0.665 | (0.529–0.772) | 4.30  mg/L | 0.32 | (0.172–0.516) | 0.98 | (0.919–0.994) | 0.80 | (0.490–0.943) | 0.83 | (0.747–0.892) | 8 | 2 | 84 | 17 |
| ***WBC*** | 144 | 0.616 | (0.508–0.725) | 15.00  x10^9/L | 0.53 | (0.364–0.691) | 0.71 | (0.615–0.782) | 0.34 | (0.224–0.478) | 0.84 | (0.753–0.901) | 17 | 33 | 79 | 15 |
| ***PRE*** | 101 | 0.570 | (0.444–0.680) | 262.00  ng/L | 0.79 | (0.595–0.908) | 0.48 | (0.373–0.590) | 0.32 | (0.217–0.449) | 0.88 | (0.750–0.948) | 19 | 40 | 37 | 5 |

Abbreviations: AUC, area under the curve; TP, true positives; FP, false positives; TN, true negatives; FN, false negatives; PPV, positive predictive value; NPV, negative predictive value; ICIS, Intensive Care Infection Score; CRP, C-reactive protein; PCT, procalcitonin; WBC, white blood cell count; PRE, presepsin; CAL, calprotectin; LBP, lipopolysaccharide-binding protein.

**Supplementary Table S2.** Comparison of biomarker levels between infected and non-infected observations

| ***Marker*** | ***N***  ***(Infected)*** | ***N***  ***(Non-infected)*** | ***Median level (Infected)*** | ***Median level***  ***(Non-infected)*** | ***U*** | ***p-value*** | ***q-value*** |
| --- | --- | --- | --- | --- | --- | --- | --- |
| ***ICIS*** | 32 | 112 | 8.00 | 2.00 | 3317.5 | 1.42e-13 | 9.95e-13 |
| ***CRP*** | 32 | 113 | 20.20 mg/L | 4.00 mg/L | 2565.5 | 0.000179 | 0.000625 |
| ***PCT*** | 30 | 98 | 0.23 µg/L | 0.08 µg/L | 2114.0 | 0.000288 | 0.000672 |
| ***LBP*** | 13 | 38 | 20.20 µg/L | 10.60 µg/L | 362.5 | 0.0129 | 0.0181 |
| ***CAL*** | 25 | 86 | 2.17 mg/L | 1.50 mg/L | 1430.5 | 0.0122 | 0.0181 |
| ***WBC*** | 32 | 112 | 15.10 x 10^9/L | 12.04 x 10^9/L | 2208.0 | 0.0458 | 0.0535 |
| ***PRE*** | 24 | 77 | 318.00 ng/L | 279.00 ng/L | 1053.5 | 0.303 | 0.303 |

ICIS, Intensive Care Infection Score; CRP, C-reactive protein; PCT, procalcitonin; WBC, white blood cell count; PRE, presepsin; CAL, calprotectin; LBP, lipopolysaccharide-binding protein.

**Supplementary Table S3.** Kendall’s tau-b correlations between biomarkers with FDR correction

| ***Biomarker 1*** | ***Biomarker 2*** | ***N*** | ***Tau*** | ***p-value*** | ***q-value*** |
| --- | --- | --- | --- | --- | --- |
| PCT | CRP | 128 | 0.447 | 8.41e-12 | 1.77e-10 |
| LBP | PCT | 51 | 0.487 | 7.1e-07 | 4.97e-06 |
| LBP | CRP | 51 | 0.509 | 6.01e-07 | 4.97e-06 |
| CAL | ICIS | 110 | 0.313 | 4.58e-06 | 2.4e-05 |
| ICIS | WBC | 143 | 0.263 | 1.08e-05 | 4.52e-05 |
| PCT | ICIS | 127 | 0.257 | 6.51e-05 | 0.000228 |
| CAL | WBC | 110 | 0.243 | 0.000199 | 0.000597 |
| PCT | PRE | 99 | 0.252 | 0.000295 | 0.000774 |
| PCT | CAL | 109 | 0.226 | 0.000701 | 0.00164 |
| LBP | WBC | 51 | -0.298 | 0.00218 | 0.00458 |
| CAL | CRP | 111 | 0.203 | 0.00393 | 0.0075 |
| PRE | CAL | 95 | 0.167 | 0.0171 | 0.0277 |
| CRP | ICIS | 144 | 0.151 | 0.0166 | 0.0277 |
| PRE | CRP | 101 | 0.163 | 0.0251 | 0.0377 |
| PRE | WBC | 100 | -0.125 | 0.0665 | 0.0931 |
| LBP | CAL | 51 | 0.168 | 0.0834 | 0.106 |
| CRP | WBC | 144 | -0.103 | 0.0856 | 0.106 |
| LBP | PRE | 36 | 0.179 | 0.127 | 0.148 |
| LBP | ICIS | 51 | 0.135 | 0.179 | 0.198 |
| PRE | ICIS | 100 | 0.066 | 0.357 | 0.374 |
| PCT | WBC | 128 | -0.019 | 0.751 | 0.751 |

ICIS, Intensive Care Infection Score; CRP, C-reactive protein; PCT, procalcitonin; WBC, white blood cell count; PRE, presepsin; CAL, calprotectin; LBP, lipopolysaccharide-binding protein.

**Supplementary Table S4.** Kendall’s tau-b correlations between biomarkers and TBSA with FDR correction

| ***Biomarker*** | ***With*** | ***N*** | ***Tau*** | ***p-value*** | ***q-value*** |
| --- | --- | --- | --- | --- | --- |
| LBP | TBSA | 51 | 0.086 | 0.39 | 0.523 |
| PCT | TBSA | 128 | 0.058 | 0.356 | 0.523 |
| PRE | TBSA | 101 | -0.111 | 0.112 | 0.523 |
| CAL | TBSA | 111 | -0.043 | 0.523 | 0.523 |
| CRP | TBSA | 145 | 0.079 | 0.203 | 0.523 |
| ICIS | TBSA | 144 | 0.039 | 0.522 | 0.523 |
| WBC | TBSA | 144 | 0.069 | 0.24 | 0.523 |

ICIS, Intensive Care Infection Score; CRP, C-reactive protein; PCT, procalcitonin; WBC, white blood cell count; PRE, presepsin; CAL, calprotectin; LBP, lipopolysaccharide-binding protein. TBSA, Total Body Surface Area.

**Figure S1.** Correlation among biomarkers and TBSA


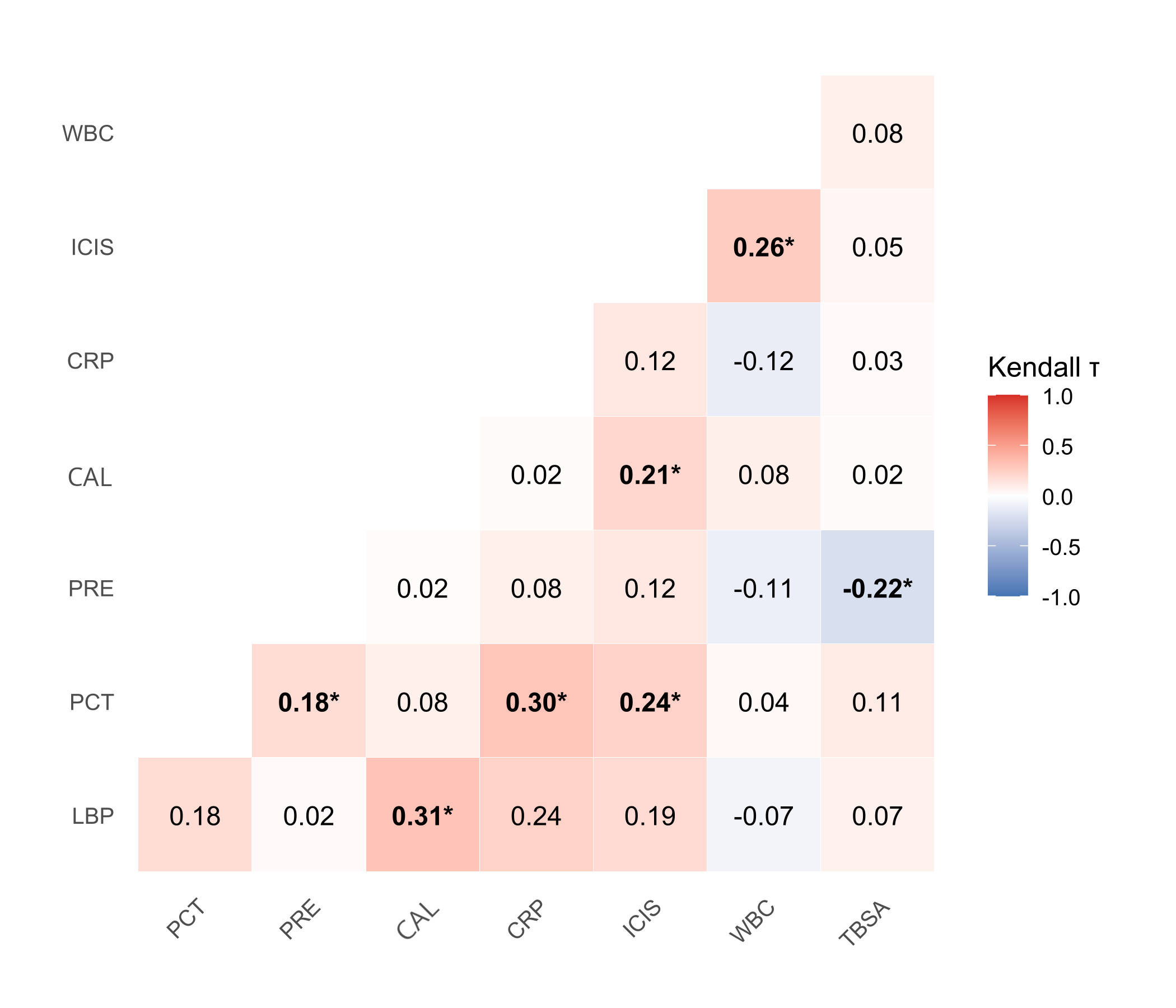


WBC, white blood cell count; ICIS, Intensive Care Infection Score; CRP, C-reactive protein; CAL, calprotectin; PRE, presepsin; PCT, procalcitonin; LBP, lipopolysaccharide-binding protein; TBSA, Total Body Surface Area

**Supplementary Table S5.** Detailed overview of infectious episodes

| ***Site of infection*** | ***Pathogen*** | ***n*** |
| --- | --- | --- |
| **Burn wound** | *Staphylococcus* spp. | 49 |
|  | *Streptococcus* spp. | 10 |
|  | Viridans group *Streptococcus* | 8 |
|  | *Enterobacter cloacae* | 8 |
|  | *Acinetobacter* spp. | 5 |
|  | Coagulase-negative *Staphylococcus* | 2 |
|  | *Klebsiella oxytoca* | 2 |
|  | *Klebsiella pneumoniae* | 1 |
|  | *Morganella morganii* | 1 |
|  | *Stenotrophomonas maltophilia* | 1 |
| **Bloodstream** | *Staphylococcus* spp. | 7 |
|  | *Acinetobacter* spp. | 2 |
|  | *Klebsiella pneumoniae* | 1 |
|  | *Stenotrophomonas maltophilia* | 1 |
|  | *Streptococcus* spp. | 1 |
| **Respiratory tract (BAL)** | *Staphylococcus* spp. | 2 |
|  | Herpes simplex virus type 1 | 1 |
|  | *Streptococcus agalactiae* | 1 |
| **Urinary tract** | *Escherichia coli* | 5 |
|  | *Enterobacter cloacae* | 2 |
|  | *Pseudomonas* spp. | 2 |

Data are presented at the observation (culture) level. Some patients experienced infections involving more than one anatomical site.

## Supplementary Table S6. Sensitivity analysis restricted to the first sample per patient.

*Diagnostic performance of ICIS, C-reactive protein (CRP), and procalcitonin (PCT) restricted to the first available observation per patient. Data are presented at the patient level (one observation per patient).*

| ***Biomarker*** | ***n*** | ***Infected, n*** | ***AUC (95% CI)*** | ***Cut-off*** | ***Sensitivity*** | ***Specificity*** | ***PPV*** | ***NPV*** |
| --- | --- | --- | --- | --- | --- | --- | --- | --- |
| ICIS | 44 | 8 | 0.887 (0.772–0.974) | ≥4 | 1.00 | 0.61 | 0.36 | 1.00 |
| CRP, mg/L | 45 | 8 | 0.625 (0.362–0.876) | ≥18.1 | 0.25 | 1.00 | 1.00 | 0.86 |
| PCT, ng/mL | 44 | 8 | 0.536 (0.289–0.782) | ≥0.17 | 0.25 | 0.89 | 0.33 | 0.84 |

AUC indicates area under the receiver operating characteristic curve. CI, confidence interval; PPV, positive predictive value; NPV, negative predictive value.
